# The occurrence of antibiotic‐resistant bacteria on the clothes of nursery teachers in daycare centres

**DOI:** 10.1111/jam.15520

**Published:** 2022-03-21

**Authors:** Dominika Žagar, Anamarija Zore, Karmen Godič Torkar

**Affiliations:** ^1^ Department of Sanitary Engineering, Faculty of Health Sciences University of Ljubljana Ljubljana Slovenia

**Keywords:** clothes, childcare Centre, antimicrobial resistance, extended‐spectrum β‐lactamases, MRSA, public health

## Abstract

**Aims:**

Childcare facilities act as microenvironments that facilitate and promote the selection, spread and transmission of antibiotic‐resistant micro‐organisms in the community. We focused on the study of antimicrobial resistance and genetic predispositions for β‐lactamase production in bacterial isolates from nursery teachers' clothing.

**Methods and Results:**

Antimicrobial resistance of bacterial strains belonging to *Enterobacteriaceae*, *Enterococcus*, *Staphylococcus* spp., *Pseudomonas* spp. and *Bacillus* spp. isolated from 80 samples of nursery teachers' clothing was determined. The selected ESβL genes were found in 30 (44.1%) of 68 strains examined. The CTX‐M type ESβL determinants were detected in 15.4%, 71.5% and 42.5% of the *Enterobacteriaceae*, *Pseudomonas* and *Bacillus* isolates, respectively. The OXA‐type coding genes were detected only in strains of the genera *Pseudomonas* (57.1%) and *Bacillus* (48.6%). Thus, most *B. cereus* strains were sensitive to the recommended antibiotics used to treat infections caused by these bacteria. Methicillin resistance was phenotypically confirmed in 27 (14.6%) of 185 staphylococcal isolates. Four isolates (2.2%) were identified as MRSA. Vancomycin resistance was not observed in any of the staphylococcal and enterococci strains.

**Conclusions:**

This study has shown that potential pathogens have been isolated from the clothing of nursery teachers, posing a risk of transmission to children. These clothes should be maintained and properly laundered to avoid cross‐contamination and the spread of multidrug‐resistant (MDR) bacteria in childcare centres.

**Significance and impact of the study:**

This study provides insight into the route of transmission of MDR micro‐organisms through the clothing of nursery teachers, to which greater importance should be given in the future. Proper procedures for the cleaning and use of clothing in daycare centres should be clarified and standardized.

## INTRODUCTION

Child daycare centres are one of the indoor environments that may be crucial in understanding the dynamics of antimicrobial resistance transmission to humans (Mills & Lee, 2019). The health impact of environmental microbiological exposure is greater in children under the age of 5, due to their physiological immaturity, and lack of hygiene habits, such as hand washing and adequate toilet use. Children in childcare centres get more infections because they come in close contact with other children in the group; they also share toys and touch each other during playing, which spreads germs. Childcare centres contain greater indoor concentration of bacteria, when compared to schools and older adult care facilities (Madureira et al., 2015). Prussin et al. (2016) found several bacterial species from the genera *Bacillus*, *Staphylococcus*, *Brevibacillus*, *Pseudomonas*, *Moraxella*, *Enterococcus*, *Acinetobacter* and *Microbacterium* on the toys and furniture in childcare centres. Even though most of these bacteria are not primary pathogens, some can survive and be transmitted through the surfaces of indoor environments, and cause community‐acquired infections. These bacteria may be nosocomial and multidrug resistant, as in the case of methicillin‐resistant *Staphylococcus aureus* (MRSA), as well as members of the *Enterobacteriaceae* family that produce extended‐spectrum β‐lactamases (ESβL) and carbapenemases (de Carvalho et al., 2017; Mills & Lee, 2019; Soto Lesmes et al., 2020).

Several studies have demonstrated the microbial contamination of healthcare workers' uniforms and clothing during patient care activities (Abu Suliman et al., 2021; Chemaly et al., 2014). Micro‐organisms can survive on commonly used fabrics from days to months (Koca et al., 2012). With increasing levels of multidrug‐resistant (MDR) bacteria present in hospital settings, the role of environmental factors, including uniforms and the ways of their washing in the spread of infection, is being intensively examined (Chemaly et al., 2014).

However, there is very little information regarding the possible pathways of antibiotic‐resistant bacteria via clothes or uniforms in child daycare settings. The personnel can carry these micro‐organisms on their hands or clothes and transfer them to the children, as well as out of the facility into the community and their own homes. Young children need a great deal of ‘hands on’ care. Therefore, the nursery teachers and their assistants must take care of personal hygiene, including handwashing and properly maintaining their clothes or uniforms.

The aim of the current study was to evaluate the contamination of the clothing of educators in public daycare centres with some main bacterial groups. The ESβLs‐ and metallo‐β‐lactamases (MβL)‐producing strains from the family *Enterobacteriaceae* and the genus *Bacillus*, as well as the presence of methicillin‐resistant *S. aureus* (MRSA), vancomycin‐resistant *S. aureus* (VRSA) and vancomycin‐resistant enterococci (VRE) were studied in more detail.

## MATERIALS AND METHODS

### Samples and isolation of bacterial strains

The 80 samples from the surfaces of the clothing of 10 nursery teachers and their assistants were collected from two child daycare centres in Lendava city (Slovenia) twice in the winter and then again in the summer of 2019. Swabs with 10 ml of saline solution were taken from 100 cm^2^ of the work clothes at the shoulder and mid‐abdominal area according to the international standard (ISO 18593, 2018). The abdominal area is the most exposed to contamination in various activities, while children often lean on nursery teachers' shoulders when being carried or nurtured by them.

Isolation of representatives of *Enterobacteriaceae*, *Enterococcus* spp., *Staphylococcus* spp., *Pseudomonas* spp. and *Bacillus* spp. was done using the standard plate count method (ISO 4833‐1, 2013). *Escherichia coli* (*E. coli*) and other enterobacteria were detected on TBX Chromogenic Agar (Tryptone Bile X‐Glucuronide) (Biolife, Italy) after 24 h of incubation at 44°C and 36°C, respectively (ISO 16649‐2, 2001). Some strains that formed colonies on TBX medium were subsequently identified biochemically as *Pseudomonas* spp. and were also included in the study. The *Bacillus* isolates were cultivated on Mannitol Yolk Polymyxin (MYP) medium (Merck Millipore, Germany) at 30°C for 24–48 h after heat treatment of samples at 80°C for 10 min to kill the vegetative cells and activate sporulation (ISO 7932, 2004). The presence of enterococci was detected on KF Streptococcus Agar Base supplemented with TTC reagent (Merck, Germany) after 44 h incubation at 37°C. Then, the obtained isolates were confirmed on Bile Esculin Azide agar (Merck Millipore, Germany). Staphylococcal isolation was carried out using Baird Parker medium (Merck Millipore, Germany) with Rabbit plasma fibrinogen supplement RPF (05939, Merck Millipore, Germany) and after 24 h of incubation at 37°C (ISO 6888‐2, 1999).

Typical colonies from each medium were prepared as pure bacterial cultures and confirmed with Gram staining microscopy, catalase and oxidase production, haemolytic activity and biochemical assays API 10S, API Staph and API 50 CH using API WEB identification programme V2.1 (bioMerieux) according to the manufacturer’s instructions. In addition, all strains presumed to belong to the genus *Staphylococcus* were confirmed by Matrix‐assisted laser desorption/ionization time‐of‐flight mass spectrometry (MALDI‐TOF MS) (Bruker MALDI Biotyper® CA System). Additional identification and classification of presumptive *Bacillus cereus/thuringiensis* strains was performed by multiplex PCR according to Park et al. (2007). The specific oligonucleotide primers and reaction parameters are shown in Table 1.

**TABLE 1:** Oligonucleotide primers used for *B. cereus* group identification, detection of β‐lactamase and *AmpC* genes in the *Enterobacteriaceae, pseudomonas* spp. and *bacillus* spp. strains

Target sequence	Nucleotide sequence (5′ → 3′)	Orientation	Designation	Amplicon’s expected size (bp)	Reference	PCR conditions
*bla*_*TEM*_	ATG AGT ATT CAA CAT TTC CG	F	OT‐3	850	Arlet et al. (1995)	94°C/3 min; 35 cycles 94°C/30 s, 55°C/30 s, 72°C/45 s; 72°C/5 min
	CCA ATG CTT AAT CAG TGA GG	R	OT‐4			
*bla*_*CTX‐M*_ *consensus*	SCS ATG TGC AGY ACC AGT AA	F	MA‐1	554	Woodford (2010)	
	CCG CRA TAT GRT TGG TGG TG	R	MA‐2			
*bla*_CTX‐M1_	AAA AAT CAC TGC GCC AGT TC	F		415	Woodford (2010)	
	AGC TTA TTC ATC GCC ACG TT	R				94°C/5 min; 30 cycles 94°C/25 s, 52°C/40 s, 72°C/50 s; 72°C/6 min
*bla*_CTX‐M2_	CGA CGC TAC CCC TGC TAT T	F		552		
	CCA GCG TCA GAT TTT TCA GG	R				
*bla*_CTX‐M8_	TCG CGT TAA GCG GAT GAT GC	F		666		
	AAC CCA CGA TGT GGG TAG C	R				
*bla*_CTX‐M9_	CAA AGA GAG TGC AAC GGA TG	F		205		
	ATT GGA AAG CGT TCA TCA CC	R				
*bla*_CTX‐M25_	GCA CGA TGA CAT TCG GG	F		327		
	AAC CCA CGA TGT GGG TAG C	R				
*bla*_IMP_	GGA ATA GAG TGG CTT AAT TCT C	F		188		
	CCA AAC CAC TAC GTT ATC T	R				
*bla*_VIM_	GAT GGT GTT TGG TCG CAT A	F		390		
	CGA ATG CGC AGC ACC AG	R				
*bla*_GIM_	TCG ACA CAC CTT GGT CTG AA	F		477		
	AAC TTC CAA CTT TGC CAT GC	R				
*bla*_SPM_	AAA ATC TGG GTA CGC AAA CG	F		271		
	ACA TTA TCC GCT GGA ACA GG	R				
*bla*_SIM_	TAC AAG GGA TTC GGC ATC G	F		570		
	TAA TGG CCT GTT CCC ATG TG	R				
*bla*_oxa‐51_	TAA TGC TTT GAT CGG CCT TG	F		353	Woodford (2010)	94°C/5 min; 30 cycles 94°C/25 s, 52°C/40 s, 72°C/50 s; 72°C/6 min
	TGG ATT GCA CTT CAT CTT GG	R				
*bla*_oxa‐23_	GAT CGG ATT GGA GAA CCA GA	F		501		
	ATT TCT GAC CGC ATT TCC AT	R				
*bla*_oxa‐40_	GGT TAG TTG GCC CCC TTA AA	F		246		
	AGT TGA GCG AAA AGG GGA TT	R				
*bla*_oxa‐58_	AAG TAT TGG GGC TTG TGC TG	F		599		
	CCC CTC TGC GCT CTA CAT AC	R				
*bla*_oxa‐48_	GCTTGATCGCCCTCGATT GATTTGCTCCGTGGCCGAAA	F R	*Multi*_*OXA‐48*_	281	Dallenne et al. (2010)	94°C/10 min; 30 cycles 94°C/40 s, 60°C/40 s, 72°C/1 min; 72°C/7 min
*AmpC*	GCTGCTCAAGGAGCACAGGAT	F	*MOXMF*	520	Manoharan et al. (2012)	
	CACATTGACATAGGTGTGGTGC	R	*MOXMR*			95°C/2 min; 30 cycles 94°C/45 s, 62°C/45 s, 72°C/1 min; 72°C/5 min
	TGGCCAGAACTGACAGGCAAA	F	*CITMF*	462		
	TTTCTCCTGAACGTGGCTGGC	R	*CITMR*			
	AACTTTCACAGGTGTGCTGGGT	F	*DHAMF*	405		
	CCGTACGCATACTGGCTTTGC	R	*DHAMR*			
	AACAGCCTCAGCAGCCGGTTA	F	*ACCMF*	346		
	TTCGCCCGAATCATCCCTAGC	R	*ACCMR*			
	TCGGTAAAGCCGATGTTGCGG	F	*EBCMF*	302		
	CTTCAACTGCGGCTGCCAGTT	R	*EBCMR*			
	AACATGGGGTATCAGGGAGATG	F	*FOXMF*	190		
	CAAAGCGCGTAACCGGATTGG	R	*FOXMR*			
*Bla*I	GTGGATGAAAGGAAATGCTACG	F		156	Wagner et al. (2011); Hussain *et al*. (1987); Madgwick & Waley (1987)	94°C/5 min; 35 cycles 94°C/30 s, 55°C/1 min, 72°C/1 min; 72°C/5 min
	ATTGCGATGATAATTGGTGCTC	R				
*Bla*II	GCGTCAGCACATTCTCAATCG	F		165		
	ATCCAGGGAAAGGACATACAGAAG	R				
*Bla*III	TGCGGGTGTACCAAAAGGATGGG	F		464		
	GGGTGGTGCAAGTGGGAAAGCAA	R				
*B. cereus group*	GTGCGAACCCAATGGGTCTTC	F	*BCGSHF*	400	Park et al. (2007)	94°C/5 min; 30 cycles 94°C/30 s, 63°C/30 s, 72°C/30 s; 72°C/5 min
*groEL*	CCTTGTTGTACCACTTGCTC	R	*BCGSHR*			
*B. anthracis*	GGTAGATTAGCAGATTGCTCTTCAAAAGA	F	*BASH2F*	253		
*gyrB*	ACGAGCTTTCTCAATATCAAAATCTCCGC	R	*BASH2R*			
*B. thuringiensis*	GCTTACCAGGGAAATTGGCAG	F	*BTJH1F*	299		
*gyrB*	ATCAACGTCGGCGTCGG	R	*BTJHR*			
*B. cereus*	TCATGAAGAGCCTGTGTACG	F	*BCJHF*	475		
*gyrB*	CGACGTGTCAATTCACGCGC	R	*BCJH1R*			
*B. mycoides*	TTTTAAGACTGCTCTAACACGTGTAAT	F	*BMSHF*	604		
*gyrB*	TTCAATAGCAAAATCCCCACCAAT	R	*BMSHR*			

### Susceptibility testing

The antimicrobial susceptibility of isolates was determined using the Kirby‐Bauer disc diffusion method on Muller‐Hinton agar (MHA, Merck Millipore, Germany) or the microdilution method according to the Clinical and Laboratory Standards (CLSI, 2016; CLSI, 2017). The control strains used to validate the antimicrobial susceptibility testing were *E. coli* ATCC 25922, *Klebsiella pneumoniae* (*K. pneumoniae*) ATCC 700603 and *Staphylococcus aureus* (*S. aureus*) ATCC 25923 (CLSI, 2017).

Phenotypic confirmation of ESβL and carbapenemase production in 26 enterobacteria, 7 *Pseudomonas* spp. and 35 *B. cereus*/*thuringiensis* strains was performed using HiCrome™ ESβL Agar (Himedia Laboratories, India) and a combined disc assay based on reduction of inhibition zones around antibiotic discs by clavulanic acid (CA) (CLSI, 2017). For this purpose, the following antibiotic discs were used: cefotaxime (30 μg), cefpodoxime (10 μg), ceftazidime (30 μg), cefepime (30 μg), cefotaxime‐CA (30/10 μg), cefpodoxime‐CA (10/1 μg), cefepime‐CA (30/10 μg) and ceftazidime‐CA (30/10 μg) (Mast Diagnostics and BBL Becton Dickinson). MβLs were detected using imipenem and meropenem discs (10 μg each) (Mast Diagnostics) combined with the same discs with added 10 μl of 0.5 moL/L EDTA (pH = 8). The discs were placed 25 mm apart on an MHA plate inoculated with a 0.5 McFarland suspension of the test isolate. The plates were incubated at 35°C for 18 h in an ambient atmosphere. Augmentation of the inhibition zone of ≥5 mm in the presence of clavulanic acid or EDTA indicates the production of ESβL or MBLs, respectively (CLSI, 2017).

The susceptibility of 35 *Bacillus cereus*/*thuringiensis* isolates was also tested for oxacillin (OX 1 μg), ciprofloxacin (CIP 5 μg), linezolid (LZD 30 μg) and gentamicin (GEN 10 μg), which are the most commonly used antibiotics for the treatment of clinical infections caused by *B. cereus* (Bottone, 2010; Ikeda et al., 2015) and interpreted according to the criteria for *Staphylococcus* spp. Minimum inhibitory concentration (MIC) values for tetracycline and vancomycin were determined and interpreted using E test strips (bioMerieux) (CLSI, 2016; CLSI, 2017).

Since *B. cereus* and *B. thuringiensis* are generally resistant to penicillins and cephalosporins due to the production of a potent broad‐spectrum β‐lactamase (CLSI, 2016), no design criteria were specified in the standards. In our case, we used the combined disc method only to determine what type of β‐lactamases these strains were likely to produce.

To predict the presence of methicillin‐resistant staphylococci, 185 colonies were picked up from Baird Parker RPF medium and transferred to HiCrome MeReSa agar (HiMedia, India). Their resistance was confirmed by disc diffusion method using cefoxitin (FOX 30 μg), while the MICs for vancomycin (European Pharmacopoeia, Merck, Germany) were determined using the microdilution method (CLSI, 2017).

The antibiotic susceptibility of 23 enterococci isolates was determined using the following antibiotic discs: vancomycin (VA 30 μg), ampicillin (AMP 10 μg), ampicillin/sulbactam (SAM 20 μg), gentamicin (GEN 120 μg) and linezolid (LZD 30 μg) (CLSI, 2017).

### Detection of genes encoding ESβLs and metallo‐carbapenemases

DNA from overnight cultures of enterobacteria strains was extracted by centrifuging, washing and boiling the cells in molecular biology‐grade water at 100°C for 10 min. Aliquots of 2 μl of template DNA were used for PCR (Queipo‐Ortuño et al., 2008). Total DNA from *Bacillus cereus*/*thuringiensis* strains was extracted using the SDS method and then purified using the phenol–chloroform–isoamyl alcohol protocol, as described previously (Sambrook et al., 1989; Mäntynen & Lindström, 1998; Moore et al., 2004). After extraction, the concentration and purity of DNA were determined using the Varian Cary 50 Bio UV–Visible Spectrophotometer (Australia).

DNA amplification with polymerase chain reaction (PCR) was performed in a 15 μl volume, consisting of 7.5 μl of DreamTaq Green PCR Master Mix (2×) (Dream Taq Polymerase, 2× DreamTaq Green Buffer, dATP, dCTP, dGTP and dTTP, 0.4 mM each, and 4 mM MgCl_2_) (Thermo Fisher Scientific), 0.4 μM of each primer and 300 ng of DNA.

Universal primers OT‐3 and OT‐4 targeting *bla*_TEM_ were used (Arlet et al., 1995). All strains were also tested using multiplex PCR with specific primers for *bla*_CTX‐M_ groups 1, 2, 8, 9 and 25. To detect variants from the families of *bla*_VIM_, *bla*_IMP_, *bla*_GIM_, *bla*_SPM‐1_, *bla*_SIM_‐_1_ and *bla*_OXA_, the multiplex primers *bla*_MBL_ VIM, IMP, GIM, SPM, SIM and OXA were used (Ellington et al., 2007; Woodford, 2010). The AmpC genes were detected according to Manoharan et al. (2012). Primers and cycling conditions are listed in Table 1. Reference strains producing CTX‐M1, CTX‐M2 and CTX‐M9 were provided by the National Institute of Biology, Ljubljana, Slovenia, while CTX‐M15‐, TEM‐1‐, VIM‐2‐ and IMP‐1‐lactamases were provided by Laboratory for Clinical and Molecular Microbiology, Clinical Hospital Center Zagreb, Zagreb, Croatia. PCR products were visualized by agarose gel electrophoresis after staining with SYBR Safe stain (Thermo Fisher Scientific).

### Detection of genes encoding BCI, BCII, BCIII at *B. cereus*/*thuringiensis* strains

The presence of *bla*I, *bla*II, *bla*III genes, encoding β‐lactamases typical for *B. cereus*, was determined using a PCR Master Kit (Roche Diagnostics, Germany). Primers for the *bla* genes of *B. cereus* species were constructed by Wagner et al. (2011) based on literature sources (Madgwick & Waley, 1987; Hussain et al., 1987) (Table 1). The standard strain *B. cereus* ATCC 14579^T^ was used as a control. PCR products were visualized by agarose gel electrophoresis after staining with SaybrSafe.

## RESULTS

### ESβLs and metallo‐carbapenemases in the isolated gram‐negative bacteria

After biochemical identification, most of the 26 isolates from the family *Enterobacteriaceae* were assigned to the genera *Serratia*, *Enterobacter*, *Klebsiella* and *Escherichia*. Only two strains, identified as *Serratia marcescens* and *E. coli*, formed colonies on HiCrome™ ESβL Agar, were resistant to all cephalosporins and meropenem tested but susceptible to imipenem, according to the standards (CLSI, 2017). Both strains were isolated from the clothes of the same nursery teacher, taken in different times of sampling. However, the inhibition zone of ≥5 mm in the presence of antibiotics with clavulanic acid or EDTA was not observed (Table 2). After PCR amplification, the *bla*_CTX‐M9_ and *bla*_OXA‐48_ genes were found in the *S. marcescens* strain, while the genes *bla*_CTX‐M1_ were detected in *E. coli* and in two other *Enterobacter* strains. The genes for *bla*_TEM_, *bla*AmpC and selected carbapenemases were not detected in any of the 26 isolates tested (Table 3).

**TABLE 2:** Resistance of tested isolates from family *Enterobacteriaceae* and genera *pseudomonas*, *bacillus*, *Staphylococus* and *enterococcus* isolated from nursery teachers' clothes

The strains and antimicrobials	No. (%) of resistant strains	No. (%) strains ≥ 5 mm
***Enterobacteriaceae*** (*n* = 26)		
Cefepime	2 (7.7)	
Cefepime/CA		0 (0)
Ceftazidime	2 (7.7)	
Ceftazidime/CA		0 (0)
Cefotaxime	2 (7.7)	
Cefotaxime/CA		0 (0)
Cefpodoxime	2 (7.7)	
Cefpodoxime/CA		0 (0)
Imipenem	0 (0)	
Imipenem/EDTA		0 (0)
Meropenem	2 (7.7)	
Meropenem/EDTA		0 (0)
*Pseudomonas* spp.^a^ (*n* = 7)		
Cefepime	7 (100)	
Cefepime/CA		0 (0)
Ceftazidime	7 (100)	
Ceftazidime/CA		0 (0)
Cefotaxime	ND	
Cefotaxime/CA		0 (0)
Cefpodoxime	ND	
Cefpodoxime/CA		0 (0)
Imipenem	5 (71.4)	
Imipenem/EDTA		0 (0)
Meropenem	2 (28.6)	
Meropenem/EDTA		0 (0)
*Bacillus s*pp. (*n* = 35)		
Cefepime^b^	ND	
Cefepime/CA		0 (0)
Ceftazidime^b^	ND	
Ceftazidime/CA		7 (20)
Cefotaxime^b^	ND	
Cefotaxime/CA		3 (8.6)
Cefpodoxime^b^	ND	
Cefpodoxime/CA		0 (0)
Imipenem^b^	ND	
Imipenem/EDTA		0 (0)
Meropenem^b^	ND	
Meropenem/EDTA		0 (0)
Oxacillin	35 (100)	
Linezolid	0 (0)	
Gentamycin	0 (0)	
Tetracycline	0 (0)	
Vancomycin	3 (8.6)	
Ciprofloxacin	0 (0)	
*Staphylococcus* spp. (*n* = 185)		
MeReSa medium^c^	27 (14.6%)	
Cefoxitin	27 (14.6%)	
Vancomycin	0 (0)	
*Enterococcus* spp. (*n* = 23)		
Ampicillin	3 (13.0)	
Ampicillin/S^d^	2 (8.7)	1 (4.3)
Gentamicin	0 (0)	
Linezolid	0 (0)	
Vancomycin	0 (0)	

Abbreviations: CA, clavulanic acid; S, sulbactam; ND, not defined.

^a^ Growth on MeReSa medium, supplemented with methicillin and cefoxitin.

^b^ *B. cereus* and *B. thuringiensis* are generally resistant to penicillins and cephalosporins due to production of a potent broad‐spectrum β‐lactamase (CLSI, 2016); there were set no interpretive criteria in standards.

^c^ The number (%) of strains, where was a difference between the zone diameters of either of the antibiotic discs and their respective antibiotic/CA or /EDTA discs for ESBLs ≥5 mm.

^d^ Andrews (2005).

^e^ The breakpoints for *P. aeruginosa* were taken into account, because in CLSI standards (2017) there were no interpretative criteria for other representatives of this genus.

**TABLE 3:** The number (%) of tested strains from fam. *Enterobacteriaceae*, genera *pseudomonas* and *bacillus* that have β‐lactamase genes

Target sequences	The number (%) of strains with target sequences for β‐lactamase genes		
	*Enterobacteriaceae* (*n* = 26)	*Pseudomonas* spp. (*n* = 7)	*Bacillus* spp. (*n* = 35)
*bla*_TEM_	0 (0)	1 (14.3)	0 (0)
*bla*_CTX‐M_ consensus	4 (15.4)	5 (71.5)	15 (42.8)
*bla*_CTX‐M1_	3 (11.5)	2 (28.6)	10 (28.5)
*bla*_CTX‐M2_	0 (0)	0 (0)	3 (8.6)
*bla*_CTX‐M8_	0 (0)	1 (14.3)	5 (14.3)
*bla*_CTX‐M9_	1 (3.8)	2 (28.6)	2 (5.7)
*bla*_CTX‐M25_	0 (0)	2 (28.6)	7 (20.0)
*bla*_IMP_	0 (0)	0 (0)	0 (0)
*bla*_VIM_	0 (0)	2 (28.6)	0 (0)
*bla*_GIM_	0 (0)	0 (0)	0 (0)
*bla*_SPM_	0 (0)	0 (0)	0 (0)
*bla*_SIM_	0 (0)	0 (0)	0 (0)
*bla*_OXA‐23_	0 (0)	1 (14.3)	2 (5.7)
*bla*_OXA‐40_	0 (0)	2 (28.6)	10 (28.5)
*bla*_OXA‐48_	1 (3.8)	3 (42.9)	5 (14.3)
*bla*_OXA‐51_	0 (0)	0 (0)	7 (20.0)
*bla*_OXA‐58_	0 (0)	0 (0)	2 (5.7)
*AmpC*	0 (0)	2 (28.6)	0 (0)

Some greenish‐beige colonies that grew on the TBX medium were identified as representatives of the genus *Pseudomonas* (*P. aeruginosa* or *P. putida*). All seven strains grown on HiCrome™ ESβL agar were resistant to cephalosporins, but the differences in the inhibition after adding CA were detected in only three of them. Resistance to imipenem and meropenem was detected in five and two strains, respectively, with no changes of inhibition zone in the presence of EDTA (Table 2). Amplifications of *bla*_CTX_ (M1, M8, M9 and M25) and *bla*_OXA_ groups (mostly OXA 40, followed by OXA 51 and OXA 23) were obtained in 5 (71.5%) and 4 (57.1%) of 7 strains, respectively. Almost all strains contained one to three CTX genes and one or two OXA genes simultaneously. The genes for *bla*_VIM_ carbapenemase and AmpC β‐lactamase were yielded in two strains, while only one strain possessed the *bla*_TEM_ type (Table 3).

### Susceptibility and the presence of genes encoding β‐lactamases in *Bacillus cereus* isolates

All 35 *B. cereus*/*thuringiensis* strains were resistant to oxacillin and susceptible to linezolid, gentamicin and tetracycline. More heterogeneous results were obtained using vancomycin with 3 (8.6%) non‐susceptible and 32 (91.4%) susceptible isolates, while 33 (94.3%) and only two (5.7%) isolates were susceptible and intermediate to ciprofloxacin, respectively. Phenotypic evidence of ESβL production was revealed in 10 (28.6%) *B. cereus*/*thuringiensis* isolates. The inhibition zone of ≥5 mm in the presence of clavulanic acid was obtained around ceftazidime and cefotaxime discs in 7 (20%) and 3 (8.6%) strains, respectively. None of the strains tested showed the production of presumptive carbapenemases after the addition of EDTA to the imipenem and meropenem discs (Table 2).

The sequences for ESβLs were found in a total of 21 (60%) of the strains, of which *bla*_CTX‐M_ type was detected in 15 (42.8%) and *bla*_OXA_‐type in 17 (48.6%). The genes for *bla*_TEM_, AmpC and selected carbapenemases were not detected in any of the strains studied. All strains with *bla*_OXA_ were also positive for one or more *bla*_CTX_ genes simultaneously. Two or more different *bla*_CTX_ and *bla*_OXA_ sequences were characteristic of 6 (17.1%) and 11 (31.4%) of 35 isolates, respectively (Table 3). ESβL‐positive strains were isolated from the clothing of 2 (20%) of 10 nursery teachers and their assistants.

### Phenotypic characterization of methicillin and vancomycin resistance in staphylococci

Methicillin resistance was confirmed on chromogenic agar medium in 27 (14.6%) out of 185 presumptive staphylococci strains. These isolates were identified as *S. aureus* (*n* = 4, 14.8%) and coagulase‐negative staphylococci (CoNS) (*n* = 23, 85.2%). MRSA thus represents 2.2% of all 185 strains examined. Among CoNS, 9 (33.3%) strains belonged to the species *S. warneri*, followed by *S. epidermidis*, *S. lugdunensis* and *S. hominis*, as well as to some other representatives of the genus. All *S. aureus* and CoNS, grown on HiCrome MeReSa agar, were resistant to cefoxitin according to zone diameter interpretation criteria (CLSI, 2017). Ten (37%) of 27 strains were susceptible to vancomycin (MIC ≤2 μg/ml), and 63% of them showed intermediate susceptibility (MIC 4–8 μg/ml) (CLSI, 2017), so we did not confirm the VRSA or vancomycin‐resistant CoNS (VRS) in any case (Table 2). Methicillin‐resistant CoNS (MRS) and MRSA positive strains were isolated from the clothing of 3 (33.3%) and 2 (20%) of 10 nursery teachers and their assistants, respectively.

### Antibiotic susceptibility of enterococci

Of 23 strains of the genus *Enterococcus* (mainly *E. faecalis* and *E. faecium*), all were susceptible to gentamicin, linezolid and vancomycin. Resistance to ampicillin was detected in 3 (13.0%) of the strains, all of which belonged to *E. faecium*. β‐lactamase inhibition by sulbactam was confirmed in one strain (Table 2).

## DISCUSSION

To cut costs, more and more hospitals are allowing staff to launder their uniforms at home. However, the results of Chiereghin et al. (2020) indicate that none of the naturally contaminated uniforms worn by healthcare workers was completely decontaminated after any of the home washes.

There is little information on the transmission of infections through the clothing of professional staff in child daycare centres. In these facilities, representatives of *Enterobacteriaceae*, *S. aureus*, *P. aeruginosa*, *Bacillus* spp., *Proteus* spp., micrococci and enterococci were the most frequently isolated bacteria from the air, toys, floors, cots, toilets and hands of the nursery teachers and their assistants (Ali et al., 2018).

We focused on the study of genetic predispositions for the production of β‐lactamases in isolates from nursery teachers' uniforms, belonging to *Enterobacteriaceae*, *Pseudomonas* and *Bacillus* species. Resistance of staphylococcal and enterococcal strains to methicillin and vancomycin was also determined.

The reasons for the low number of Gram‐negative strains isolated could be the good maintenance of personal hygiene. Bloomfield et al. (2011) reported that Gram‐negative pathogens such as *P. aeruginosa* and enteric bacteria such as *Salmonella*, *Shigella*, *Campylobacter*, *E. coli* were found less frequently on clothing, probably because they require a moist environment to survive.

Only four (15.3%) of 26 isolates from the *Enterobacteriaceae* family identified as *S. marcescens*, *E. coli* and *Enterobacter* spp. yielded genes for ESβLs, despite negative results by the phenotypic combined disc method. *S. marcescens* causes both opportunistic and nosocomial infections. It can survive in the presence of disinfectants, antiseptics and on various surfaces in clinical departments (Godič Torkar & Ivić, 2017). All *Serratia* species are intrinsically sensitive to carbapenems, although some *S. marcescens* strains have been identified to harbour both chromosomal and plasmid‐mediated carbapenemase genes, such as OXA‐β‐lactamases (Evans & Amyes, 2014; Hopkins et al., 2017). Its antibiotic resistance has also been reported to penicillins, first‐ and second‐generation cephalosporins, aminoglycosides and polymyxins. *S. marcescens* strains most commonly carry CTX‐M‐type ESβLs, specifically *bla*_CTX‐1_, *bla*_CTX‐3_, *bla*_CTX‐9_, *bla*_CTX‐15_ and *bla*_OXA‐48_ carbapenemases (Hopkins et al., 2017; Regev‐Yochay et al., 2018). The *bla*_CTX‐9_ and *bla*_OXA‐48_ genes were also present in the *Serratia* strain of the current study.

Opportunistic pathogens *E. coli* and *Enterobacter* spp. are typical representatives of antimicrobial‐resistant bacteria in the community, especially in childcare settings (Furuya & Lowy, 2006). β‐Lactam resistance is usually mediated by the acquisition of β‐lactamase genes on plasmids or transposons. Most β‐lactamases found in *E. coli* belong to Ambler class A (e.g. TEM‐1, TEM‐2 and SHV‐1) and ESβLs (e.g. TEM‐3, SHV‐5 and CTX‐M‐like) (Bush et al., 1995; Tartor et al., 2021). In our study, the *E. coli* and the two *Enterobacter* strains were positive for *bla*_CTX‐M1_, which are the most common ESβLs among *E. coli* in hospitals (Pournaras et al., 2004) and also in the faeces of healthy human carriers (González et al., 2020). However, in the current study, the genes for *bla*_TEM_, *bla*_AmpC_ and selected carbapenemases were not detected in any of the 26 isolates tested, as reported by González et al. (2020).

Most species of the genus *Pseudomonas* can form biofilms on various surfaces, including textiles, and are difficult to eliminate with disinfectants and antibiotics. They have intrinsic, adaptive and acquired resistance mechanisms. Among the most important are the presence of β‐lactamases, changes in membrane permeability due to the presence of efflux pumps and mutations of transmembrane porins (Cillóniz et al., 2019). The types TEM, SHV and OXA are the most common ESβLs found in *P. aeruginosa* isolates (Chen et al., 2015; Lin et al., 2012). Class C cephalosporinases (AmpC‐β‐lactamases) are also an important cause of multiple β‐lactam resistance in *P. aeruginosa* (Mirsalehian et al., 2014). In our *P. aeruginosa/putida* isolates, *bla*_CTX_ and *bla*_OXA‐like_ (OXA 40, followed by OXA 51 and OXA 22) and AmpC genes were mostly yielded, while TEM was possessed by only one strain. Some authors reported that OXA genes in this bacterium are localized on the chromosome or transmitted by plasmids or transposons, sometimes in combination with other ESβLs (Antunes & Fisher, 2014). The simultaneous presence of OXA and CTX types was also confirmed in most of our strains. *P. aeruginosa* is intrinsically resistant to several β‐lactams, while it is generally still relatively susceptible to carbapenems (El Amin et al., 2005). The SHV type was absent in all isolates tested. We detected the *bla*_VIM_ genes in two *Pseudomonas* strains without phenotypic confirmation with EDTA, although these strains were resistant to meropenem and imipenem (Table 2). We can assume that their resistance is due to an interplay of low permeability or activity of the efflux pump and not to the production of chromosomal β‐lactamases (El Amin et al., 2005; Hammami et al., 2009).

*B. cereus* can be responsible for food poisoning and cause various infections, mainly in immunocompromised individuals. Its endospores are resistant to pasteurization, desiccation and to various disinfectants (Bottone, 2010). Cheng et al. (2017) also reported a sudden increase in invasive nosocomial infections in patients caused by *B. cereus* group organisms transmitted through improperly washed linen in hospitals. The results of our study with 100% susceptible *B. cereus* strains to tetracycline, gentamicin, linezolid, ciprofloxacin and vancomycin were consistent with previous reports (Bottone, 2010; Shawish & Tarabees, 2017), although we obtained three (8.6%) vancomycin‐non‐susceptible and two (5.7%) ciprofloxacin‐intermediate strains (Table 2). Similar results with 6.1% vancomycin‐non‐susceptible and 1.5% ciprofloxacin‐intermediate strains isolated from clinical specimens were also obtained in our previous study (Godič Torkar & Bedenič, 2018).

Phenotypic evidence of ESβL production was obtained in 28.6% of *B. cereus*/*thuringiensis* isolates (Table 2). After confirmation of ESβLs predispositions with PCR method, this percentage was even higher with a total of 60% positive strains, of which one or more *bla*_CTX‐M_‐type and *bla*_OXA_‐type genes were found in 42.8% and 48.6%, respectively. The *bla*_OXA_ genes were determined in half of the tested strains (Tables 2 and 3). The OXA‐β‐lactamases had lower specific activity against penicillin than the TEM β‐lactamases, but much higher activity against oxacillin and methicillin (Evans & Amyes, 2014). Godič Torkar and Bedenič (2018) also confirmed the amplicons of the *bla*_CTX‐M_, *bla*_VIM_ and *bla*_TEM_ genes in 68.2%, 21.2% and 34.8% of food and clinical *B. cereus* isolates, respectively. The absence of *bla*_VIM_ and *bla*_TEM_ and the lower percentage of *bla*_CTX‐M_‐positive strains (42.8%) obtained from clothes in the present study is probably the result of not being exposed to intensive antibiotic pressure therapy.

Thus, the reason for the 100% resistance of the tested strains to oxacillin is due to *B. cereus*‐specific β‐lactamases and not only to *bla*_OXA_ genes. Three different β‐lactamases, named β‐lactamase I, II and III, have been detected in this species (Chen et al., 2003). *B. cereus* serine‐β‐lactamase I (BCI) and β‐lactamase III (BCIII) are group 2A enzymes, while β‐lactamase II (BCII) is a heat‐stable, chromosomally mediated metallo‐β‐lactamase from enzyme group 3a and subclass B1 (Behravan & Rangsaaz, 2004; Bush et al., 1995; Palzkill, 2013). This molecular subclass includes IMP, VIM, NDM, DIM, GIM, SIM and SPM (Bush & Jacoby, 2010; Sawa et al., 2020). In the current study, genes from the *bla*_VIM_ *, bla*_TEM_, *bla*_SHV_, *bla*_GIM_, *bla*_IMP_, *bla*_SPM_, *bla*_SIM_ and AmpC families were not detected in the *B. cereus*/*thuringiensis* strains tested (Tables 2 and 3). Despite the expectation of intrinsic resistance to carbapenems, clinical studies show that most *B. cereus* isolates from different specimens were susceptible to carbapenems such as imipenem and meropenem, although resistant strains were also isolated (Ikeda et al., 2015). BcII genes were detected in only 77% of *B. cereus* strains isolated from nursery teachers' clothing (Table 3), while these genes were yielded in all isolates tested in a previous study (Godič Torkar & Bedenič, 2018). The same authors reported that the PCR products for *bla*I and *bla*III were rare, as was also the case in this study. It can be assumed that chromosomally encoded BCII inactivate penicillins but not carbapenems, and resistance to these antibiotics is probably a consequence of acquired genes.

Phenotypic evidence of β‐lactamase production was obtained in 23.5% of all 68 *Bacillus* spp., *Pseudomonas* spp. and *Enterobacteriaceae* strains tested, the target sequences for selected β‐lactamases and AmpC were amplified in 39.7% of them. These discrepancies in results could be due to the lower sensitivity and specificity of the phenotypic tests (Giriyapur et al., 2011). Positive phenotypic results without genotypic confirmation observed in 14.7% of cases are probably due to the fact that genes for some ESβLs (e.g. SHV, VEB, PER) (Tartor et al., 2021) were not determined in our study.

Enterococci and staphylococci survived well on common hospital linen, and their transmission from clothing via hands to surfaces in the clinical environment was also confirmed (Kanwar et al., 2018). *S. aureus* as the main representative of coagulase‐positive staphylococci and some coagulase‐negative staphylococci (CoNS) are common members of the resident and transient skin microbiota. As a group of major nosocomial pathogens, they are known to be resistant to multiple antibiotics as they possess several acquired resistance genes. The *bla*Z gene is the most common β‐lactam (penicillinase) resistance mechanism for *S. aureus*, while methicillin, clindamycin/macrolides and vancomycin resistance is mediated by the *mec*, *erm*B and *van*A genes, respectively (Akpaka et al., 2017; Emaneini et al., 2013).

Community‐associated MRSA (CA‐MRSA) has caused increased MRSA infections in community settings, including daycare centres (Bloomfield et al., 2007; Miller et al., 2011).

To detect methicillin‐resistant coagulase‐negative staphylococci (MRS) and MRSA, we used the diffusion method with cefoxitin discs in addition to the chromogenic medium. Specifically, Anand et al. (2009) confirmed a good correlation between the results obtained by the disc diffusion method and the presence of the *mec*A gene by PCR. In all, 27 (14.6%) of 185 staphylococcal isolates were grown on the chromogenic medium. Four isolates (2.2%) were identified as *S. aureus*. As expected, all of these strains were resistant to cefoxitin by the disc diffusion method (Table 2). In addition, MRS‐ and MRSA‐positive strains were isolated from the clothing of 3 (33.3%) and 2 (20%) of 10 nursery teachers and their assistants, respectively. These two MRSA‐positive individuals were employed in two different departments of the daycare centre, so we assumed that they were colonized with *S. aureus* and that the bacterium was not transmitted to their clothing from the children. These findings are similar to those reported by Moritz et al. (2015), where the prevalence of *S. aureus* among staff in daycare centres was 34.5%, while the prevalence of MRSA was 3.7%. The higher percentage of MRSA‐positive individuals is likely due to the small number of nursery teachers tested (swabs were taken twice from the same individual in each season), which is one of the shortcomings of our study.

Although we did not obtain vancomycin‐resistant *S. aureus* and CoNS, it is of concern that a large proportion (63%) of them had intermediate vancomycin susceptibility (Table 2). In contrast, only one of 70 *S. aureus* strains isolated from the nasal secretions of healthy children in Brazilian daycare centres showed intermediate resistance to vancomycin (de Carvalho et al., 2017).

Enterococci are considered opportunistic pathogens that are difficult to treat because the organisms have intrinsic and acquired resistance to many antibiotics (Yadav et al., 2017). Most of them are thermotolerant and can survive at temperatures commonly used to decontaminate healthcare linen (Orr et al., 2002). Enterococci are intrinsically resistant to some β‐lactam antibiotics such as cephalosporins and carbapenems, but these bacteria have increased resistance to penicillin and ampicillin, acquired either through mutations in penicillin‐binding proteins (PBPs) or, less commonly, through the production of β‐lactamases. While intrinsic ampicillin resistance occurs in more than 90% of *E. faecium* isolates because they encode a class B PBP with low binding affinity for ampicillin and the cephalosporins (Miller et al., 2014: Pfaller et al., 2019; López‐Luis et al., 2021), *E. faecalis* strains have lower intrinsic tolerance to ampicillin. Ampicillin resistance mediated by β‐lactamase activity has been described in both *E. faecalis* and *E. faecium* (Miller et al., 2014). Enterococci are also intrinsically resistant to clindamycin and most aminoglycosides (Kristich et al., 2014; Schell et al., 2020). Vancomycin resistance in *Enterococcus* spp. has increased alarmingly, with some studies reporting disturbingly high rates. Vancomycin resistance occurs primarily in *E. faecium* isolates, which often belong to clade A1 and possess the vancomycin resistance genes *van*A and *van*B (Garcia‐Migura et al., 2016; López‐Luis et al., 2021). Jannati et al. (2020) reported 11 (2.7%) and 10 (2.4%) highly gentamicin‐resistant and ampicillin‐resistant enterococci isolated from faecal specimens of healthy children, respectively. No vancomycin‐resistant enterococci were detected, as was the case in our study. We detected three ampicillin‐resistant strains (13%); however, only one of them showed an increase in the zone of inhibition around discs containing ampicillin and sulbactam by more than 5 mm, suggesting the production of β‐lactamases. The sporadic increase in linezolid resistance level in enterococci under the selection pressure is due to recombination between rRNA genes after the occurrence of the G2576U mutation (Kristich et al., 2014). All isolates from the educators' clothing in the present study were sensitive to linezolid, which was expected; hence, the percentage of resistant clinical isolates in other studies was also quite low (from 2% to 9%) (Klare et al., 2015; Yadav et al., 2017).

The resistant strains of the *Pseudomonas* spp., *Bacillus* spp., *Staphylococcus* spp., *Enterococcus* spp. and *Enterobacteriaceae* were isolated from the mid‐abdominal area of teachers' clothing in 85% of the cases, with the remainder found on the shoulders. These results were to be expected, as this area is one of the most exposed to contamination during the activity of the teachers in the daycare centres. No difference was observed between the ratio of resistant strains isolated during winter and summer sampling. ESβL strains were most frequently isolated from clothing belonging to teachers #1 and #6, while resistant staphylococci were isolated on clothing belonging to teachers #5 and #7. The apparent differences in contamination with resistant strains between teachers were not confirmed.

Community‐acquired MDR potential pathogens can also be spread in childcare centres, not only through direct or indirect contact via hands, food, toys or contact surfaces but also through nursery teachers' clothing. Washing clothes at home under inadequate conditions does not provide adequate hygiene standards. In addition, the colonization of healthy individuals with clinically important MDR bacteria could serve as a reservoir for the maintenance and spread of resistant strains in the environment and social settings.

## CONFLICT OF INTEREST

The authors declare no conflict of interest.

## Data Availability

Data available on request from the authors
